# Blood pressure management to prevent recurrent stroke: current evidence and perspectives

**DOI:** 10.1038/s44325-024-00021-x

**Published:** 2024-09-10

**Authors:** Gisele Sampaio Silva, João Brainer Clares de Andrade, Eduardo Bello Martins, Karla Santo, M. Julia Machline-Carrion

**Affiliations:** 1 Hospital Israelita Albert Einstein, 755 Comendador Elias Jafet Street, room 408/409, Floor L4, Morumbi, São Paulo, SP Postal Code 05653-000 Brazil; 2 Universidade Federal de São Paulo, Escola Paulista de Medicina, São Paulo, Brazil; 3 Aeronautics Institute of Technology, Sao Jose dos Campos, Brazil; 4 epHealth UK, C/O Taylor Vinters, Floor 33 Tower 42, 25 Old Broad Street, London, EC2N 1HQ UK; 5 Instituto epHealth, 2302 Consolação Street, CJ 21, Room 104, Consolação, Sao Paulo, SP Postal Code 01302-001 Brazil

**Keywords:** Cardiology, Neurology

## Abstract

Hypertension is the leading risk factor for stroke, causing about 60% of cases. Effective blood pressure control is vital for preventing recurrent ischemic strokes, with studies showing mixed results. Intensive control reduces cardiovascular events, as seen in the SPRINT, PROGRESS and STEP studies, while trials like RESPECT show no difference. Technological advances like AI and wearables enhance management, but challenges remain in achieving equitable control, especially for minorities.

## Introduction

Hypertension is the most important risk factor for stroke. High blood pressure significantly increases the risk of both initial and subsequent strokes, with hypertension contributing to approximately 60% of the risk for cerebrovascular disease across populations^1^. In the InterStroke study, individuals with hypertension were over twice as likely to suffer a stroke – in a context of primary prevention^2^. Thus, blood pressure control following a stroke underscores a critical component of secondary prevention strategies to reduce the risk of recurrent cerebrovascular events. Current evidence states the significant benefits of managing hypertension in stroke survivors, with some studies suggesting that stringent blood pressure control can substantially decrease the likelihood of subsequent strokes and other cardiovascular complications^3^. In summary, blood pressure reduction in the chronic stage of stroke is highly effective in preventing recurrent strokes, protecting cardiovascular health, preserving cognitive function, and reducing overall mortality.

Managing blood pressure after a stroke, however, presents several challenges, but it remains a cornerstone of post-stroke care and management. Adherence to medication and lifestyle modifications can be problematic, particularly in patients with cognitive impairment or other disabilities resulting from stroke. In low- and middle-income countries (LMICs), managing hypertension effectively can be particularly challenging, as exemplified by rural areas in India where access to healthcare providers and essential medications is limited, leading to high rates of uncontrolled blood pressure and subsequent cardiovascular complications^4^. Healthcare systems also face logistical hurdles in ensuring consistent follow-up and monitoring. Socioeconomic factors might further complicate effective blood pressure management in stroke survivors^5^.

We aimed to review, in a narrative perspective, the current evidence about the long-term blood pressure management for either primary or secondary Stroke prevention.

## Long-term blood pressure management to prevent recurrent stroke – where do we stand?

The Systolic Blood Pressure Intervention Trial (SPRINT) has played a pivotal role in illustrating the critical importance of lower blood pressure levels for the prevention of major cardiovascular events in high cardiovascular risk patients. By enrolling 9361 participants without diabetes and without a history of stroke, SPRINT provided clear evidence that an intensive blood pressure management strategy targeting a systolic blood pressure (SBP) below 120 mmHg significantly reduces the risk of severe cardiovascular outcomes. Specifically, the trial found a 25% reduction in the risk of acute myocardial infarction, coronary disease, stroke, heart failure, and death from cardiovascular causes among those in the intensive treatment group compared to those receiving standard treatment^6^. Another clinical study demonstrating the benefit of an intensive SBP control was the STEP. The STEP randomized 9624 elderly Chinese patients from 60 to 80-year-old to a SBP target of 110–130 mmHg or 130–150 mmHg and found a 26% risk reduction of stoke, acute coronary syndrome, acute heart failure, coronary revascularization, atrial fibrillation or cardiovascular death in the intensive arm (mean SBP of 126.7 mmHg versus 135.9 mmHg). Of note, a 33% reduction in stroke was found in STEP^7^. The SPRINT and STEP study results’ hold significant implications for clinical practices regarding blood pressure management in patients with elevated cardiovascular risk. However, because the study excluded stroke patients, the optimal blood pressure targets for secondary stroke prevention remains to be established^8^.

The ideal blood pressure target for individuals who have experienced a stroke has not yet been established by randomised controlled trials. Observational studies and randomised controlled trials suggest that individuals who achieve blood pressure below 140 systolic and 90 diastolic tend to have improved outcomes (Table 1)^3^. However, these studies have not conclusively determined the optimal extent to which blood pressure should be reduced. Current guidelines suggest maintaining a blood pressure consistently below 140/90 mmHg for those who have suffered a cerebrovascular event^9,10^.

**Table 1:** Summary of published clinical trials in blood pressure control for Stroke prevention

Trial	Participants	Design	Follow-Up	Results
SPS3 Trial (Secondary Prevention of Small Subcortical Strokes)^2^	3020 patients > 30 years with recent lacunar stroke.	Randomized to systolic BP targets of < 140 mmHg vs. < 130 mmHg.	Mean of 3.7 years.	• Systolic BP averages were 138 and 127 mmHg respectively. • 277 endpoints occurred, including 243 ischemic strokes. • Nonsignificant reduction in stroke recurrence in the intensive group (*P* = 0.08). • Significant reduction in intracerebral hemorrhage (*P* = 0.03).
RESPECT (Recurrent Stroke Prevention Clinical Outcome)^3^	In total, 1280 patients were randomized 1:1 to BP control to less than 140/90 mmHg (standard treatment) (*n* = 640) or to less than 120/80 mmHg (intensive treatment) (*n* = 640)	Randomized clinical trial (RCT) of standard vs intensive BP control in an intent-to-treat population of patients who had a history of stroke.	Mean of 3.9 (1.5) years	• Stroke Recurrence: 91 first recurrent strokes occurred during the study. • Rate Reductions: Nonsignificant rate reductions for recurrent stroke were observed in the intensive group compared to the standard group (HR, 0.73; 95% CI, 0.49–1.11; *wew* = 0.15) • Meta-Analysis: When pooled with three previous RCTs, the meta-analysis favored intensive BP control (relative risk, 0.78; 95% CI, 0.64–0.96; *P* = 0.02) • Trend: Intensive BP lowering tended to reduce stroke recurrence. Recommendation: The updated meta-analysis supports a target BP of less than 130/80 mmHg for secondary stroke prevention
SPRINT Trial (Systolic Blood Pressure Intervention Trial)^6^	9361 patients >50 years with systolic BP 130 to 180 mmHg and increased CVD risk, without diabetes or prior stroke.	Randomized to systolic BP targets of <140 mmHg vs. <120 mmHg.	Mean of 3.26 years.	• Systolic BP averages were 121.4 and 136.2 mmHg respectively. • 319 primary endpoints in the standard group vs. 243 in the intensive group (*P* < 0.001). • Significant reduction in all-cause deaths (*P* < 0.003) and CVD deaths (*P* < 0.005) in the intensive group. • More frequent adverse events related to BP lowering in the intensive group (*P* < 0.001).
PROGRESS - Perindopril Protection Against Recurrent Stroke Study^11^	Both hypertensive and non-hypertensive patients were eligible for inclusion. The study was conducted in 172 centers from 10 countries (Australia, Belgium, China, France, Italy, Ireland, Japan, New Zealand, Sweden and the United Kingdom). About 6100 patients were randomized. Mean age of participants at study entry was 64 years, 30% of whom were female, 84% had an entry diagnosis of stroke, and the remainder had an entry diagnosis of TIA alone.	Patients were randomly assigned to treatment with the ACE inhibitor perindopril (and the diuretic indapamide for those with no definite indication or contraindication to treatment with a diuretic) versus matching placebo(s)	Mean of 3.9 year	• Treatment significantly reduced the risk of stroke and major vascular events, with a 28% reduction in stroke and a 26% reduction in major vascular events overall. These reductions were consistent across different subgroups based on age, sex, and region, with particularly notable benefits observed among younger participants and those from Asia. • The treatment was deemed safe and well-tolerated, and the absolute benefits were substantial, with an estimate that treating 20 patients for 5 years would prevent at least one major vascular event. • Blood pressure lowering effectively reduces the risk of secondary stroke, with significant benefits across various demographic groups.
PROFESS (Prevention Regimen for Effectively Avoiding Second Strokes) trial^14^	The trial included over 20,000 patients who had experienced an ischemic stroke within the previous 90 days.	Patients were randomly assigned to one of two groups: • Telmisartan Group: Patients received telmisartan, an angiotensin II receptor blocker. • Placebo Group: Patients received a placebo.	The follow-up period for the trial was approximately 2.5 years	• Stroke Recurrence: The trial found no significant difference in the recurrence of stroke between the telmisartan group and the placebo group. • Cardiovascular Events: There was also no significant difference in the incidence of major cardiovascular events between the two groups. • Blood Pressure Control: Telmisartan effectively reduced blood pressure, but this did not translate into a significant reduction in stroke recurrence or major cardiovascular events. • Safety and Tolerability: The safety profile of telmisartan was consistent with previous studies, with no unexpected adverse effects.
PODCAST (Prevention of Decline in Cognition after Stroke Trial)^15^	Subjects with recent stroke, absence of dementia, and systolic BP (SBP) 125–170 mmHg were assigned randomly to at least 6 months of intensive (target SBP < 125 mmHg) or guideline (target SBP < 140 mmHg) BP lowering.	Multicenter and partial-factorial trial	The median was 24 months (range 1–48)	• In patients with recent stroke and normal cognition, intensive treatment led to significant reductions in mean blood pressure (difference −10.6/−5.5 mmHg) compared to guideline treatment. • However, there was no significant difference in the ACE-R score between the treatments for blood pressure lowering. • The mean difference for BP lowering was −3.6 (95% CI −9.7 to 2.4). • There was no difference in rates of dementia or serious adverse events for either comparison. • Overall, intensive BP lowering were feasible and safe but did not alter cognition over two years.
PAST-BP (Prevention After Stroke -Blood Pressure)^15^	People with a history of stroke or transient ischaemic attack. Interventions Intensive systolic blood pressure target ( < 130 mmHg or 10 mmHg reduction from baseline if this was <140 mmHg) or standard target ( < 140 mmHg). Apart from the different target, patients in both arms were actively managed in the same way with regular reviews by the primary care team.	Open label randomized controlled trial. Setting 99 general practices in England, with participants recruited in 2009-11.	12 months	• The intensive target arm aimed for a systolic blood pressure below 130 mmHg, resulting in a drop of 16.1 mmHg−127.4 mmHg. • The standard arm aimed for a systolic blood pressure below 140 mmHg, resulting in a drop of 12.8 mmHg to 129.4 mmHg. • The difference between the groups was 2.9 mmHg (95% confidence interval: 0.2–5.7 mmHg; *P* = 0.03). • Targeting below 130 mmHg provided a small additional reduction in blood pressure compared to targeting below 140 mmHg. • Active management aiming for a target below 140 mmHg resulted in a clinically significant reduction in blood pressure in this population.

*BP* Blood pressure, *MI* Myocardial infarction, *CVD* Cardiovascular Risk, *ACE* Angiotensin Converting Enzyme, *RCT* Randomized clinical trial, *HR* Hazard Ratio, *CI* Confidence Interval; *ACE-R* Addenbrooke’s Cognitive Examination-Revised.

In contrast to the STEP and SPRINT trial, which excluded patients with prior stroke, there are few studies that addressed this population. The Perindopril Protection Against Recurrent Stroke Study (PROGRESS) was a rigorously designed and executed clinical trial focusing on blood pressure reduction. It included 6105 patients with previous stroke or transient ischaemic attack. Participants were randomly assigned to receive perindopril, an angiotensin-converting enzyme inhibitor, with or without indapamide, a thiazide-like diuretic, or a placebo as supplementary treatment. In the group treated with perindopril plus indapamide, blood pressure decreased by about 12/5 mmHg, leading to a significant 43% relative risk reduction for the primary endpoint of total stroke. The data further suggested that more substantial blood pressure reductions correlated with greater reductions in major vascular events^11^. The use of perindopril alone resulted in a more modest blood pressure decrease of approximately 5/3 mmHg and without a statistically significant reduction in stroke risk. Significant stroke risk reduction was only observed with the combined perindopril and indapamide treatment^12^. A crucial takeaway from PROGRESS is that more intensive blood pressure lowering is linked to more pronounced benefits in reducing major vascular events^13^.

In the Prevention Regimen for Effectively avoiding Second Strokes (PRoFESS) study, 20,332 patients with previous stroke were assigned to a receive either the angiotensin receptor blocker telmisartan at 80 mg/day or a placebo alongside standard antihypertensive therapy. This study also compared the effects of aspirin with extended-release dipyridamole versus clopidogrel. Over an average monitoring period of 2.5 years, the telmisartan group showed a mean blood pressure reduction of 3.8/2.0 mmHg, with initial between-group differences in SBP of 5.4 mmHg at one month and 4.0 mmHg at one year, indicating a gradual narrowing of this difference. The incidence of recurrent strokes was similar in the telmisartan group compared to the placebo group (8.7% vs. 9.2%; hazard ratio 0.95; 95% CI, 0.87–1.01, *p* = 0.23). The occurrence of major cardiovascular events was also similar between groups (13.5% vs. 14.4%; hazard ratio 0.94; 95% CI, 0.87–1.01), (*p* = 0.11)^14^.

The Secondary Prevention of Small Subcortical Strokes (SPS3) trial included 3020 participants with MRI-confirmed symptomatic lacunar ischemic stroke within 180 days to compare a SBP target of 130 to 149 mmHg versus a SBP < 130 mmHg. After a mean follow-up of 44 months, the primary endpoint (all strokes) was observed in 125 (2.25%) participants in the intensive SBP target group versus 152 (2.77%) participants in the standard SBP target group (HR 0.81, 95% CI 0.64–1.03, *p* = 0.08). The intensive SBP reduction strategy was associated with a reduction in haemorrhagic stroke (HR 0.37, 95% CI 0.15–0.85, *p* = 0.03). No statistically significant difference was observed between groups for other secondary outcomes, including ischemic stroke (HR 0.84, 95% CI 0.66–1.09, *p* = 0.19), myocardial infarction (HR 0.88, 95% CI 0.56–1.39, *p* = 0.59), major vascular events (HR 0.84, 95% CI 0.68–1.04, *p* = 0.1), all-cause death (HR 1.03, 95% CI 0.79–1.35, *p* = 0.82) and vascular death (HR 0.86, 95% CI 0.55–1.35, *p* = 0.52). There was also no difference in serious adverse events^15^.

The Recurrent Stroke Prevention Clinical Outcome (RESPECT) trial assessed blood pressure management in 1,280 stroke survivors who had baseline SBP between 130- and 180-mmHg or diastolic pressures between 80- and 110-mmHg. The study compared a standard treatment target of <140/90 mmHg with an intensive treatment target of < /80 mmHg. Originally intended to enrol 2000 participants, the trial was prematurely halted. Over an average follow-up period of 3.8 years, 52 strokes (2.26% per year) were reported in the standard treatment group compared to 39 strokes (1.65% per year) in the intensive treatment group. The reduction in the risk of recurrent stroke with intensive blood pressure management was similar between groups (HR 0.73, 95% CI 0.49–1.11, *p* = 0.15)^3^.

Blood pressure variability (BPV) has emerged as a significant factor in stroke prevention, with numerous studies highlighting its role in predicting stroke risk and outcomes. Several studies have identified BPV as an independent predictor of stroke. Population-based studies have confirmed the association between BPV and stroke risk. The Rotterdam Study found that visit-to-visit BPV was associated with an increased risk of hemorrhagic and unspecified strokes, independent of mean BP level^16^. In addition, the China Stroke Primary Prevention Trial demonstrated that visit-to-visit SBP variability is a significant predictor of primary stroke in hypertensive patients, independent of mean SBP levels^17^. This finding underscores the importance of monitoring BPV in addition to mean BP levels in hypertensive patients to better predict and prevent stroke. BPV has also been linked to cerebral small vessel disease, which is a major contributor to stroke and dementia. A systematic review and meta-analysis found that increased BPV is associated with a higher burden of white matter hyperintensities, a marker of cerebral small vessel disease^18^. This suggests that targeting BPV could potentially reduce the risk of stroke by mitigating the progression of small vessel disease. The effectiveness of different classes of antihypertensive drugs in reducing BPV and thereby preventing stroke has been explored. A systematic review and meta-analysis revealed that calcium-channel blockers and non-loop diuretic drugs significantly reduce interindividual variation in SBP, which is a surrogate for within-individual BPV. This reduction in BPV was associated with a lower risk of stroke^19^. Conversely, drugs like ACE inhibitors and beta-blockers were found to increase BPV, potentially diminishing their effectiveness in stroke prevention^19^. For patients with a history of stroke, reducing BPV is crucial for secondary stroke prevention. A secondary analysis of the PRoFESS trial indicated that both systolic and diastolic BPV were associated with recurrent stroke and major cardiovascular events^20^. This reinforces the importance of BPV management in patients with prior stroke to prevent recurrence and improve overall cardiovascular health^16,17,21^.

In summary, evidence from four randomized controlled trials and several meta-analyses underscores the advantage of treating patients with a history of stroke or transient ischemic attack to achieve a target blood pressure of < 130/80 mmHg. The RESPECT, SPS3 (previously described), and the smaller PAST-BP and PODCAST trials evaluated the effects of intensive blood pressure control, with SBP targets ranging from < 120 to < 130 mmHg, against standard blood pressure control (SBP targets from <140 to <150 mmHg) in individuals with prior cerebrovascular disease^3,15,22,23^. These studies found a trend towards lower rates of recurrent strokes in the groups receiving intensive treatment, although these findings were not statistically significant. However, a meta-analysis of these four trials demonstrated a significant reduction in the risk of recurrent stroke when comparing intensive and standard blood pressure targets, with a relative risk (RR) of 0.78 (95% CI, 0.64–0.96)^3^. Additionally, the largest meta-analysis to date, which included data from over 40,000 patients across 14 RCTs, found a significantly lower rate of recurrent stroke among patients who achieved an SBP of < 130 mmHg compared to those with higher SBP levels^24^.

## Blood pressure agents in secondary stroke prevention

Various antihypertensive medications, including diuretics, beta-blockers, angiotensin-converting enzyme inhibitors (ACEI), angiotensin receptor blockers (ARBs), and calcium channel blockers, have been evaluated for their efficacy in preventing vascular events (Table 2)^25^. In patients with previous stroke, ACEI, and ARBs have shown positive outcomes in randomized controlled trials and systematic reviews of such studies, as discussed in the previous session. While calcium channel blockers are commonly prescribed for managing hypertension, evidence supporting their effectiveness in preventing secondary strokes is scarce. Nonetheless, employing calcium channel blockers may be a suitable option for stroke patients who need additional treatment alternatives^26^.

**Table 2:** Summary of anti-hypertensive drugs

Class of Antihypertensive	Therapeutic Advantages	Limitations	Common Adverse Effects
Angiotensin-Converting Enzyme (ACE) Inhibitors	Enhance survival in heart failure patients, renal protection, prevention of recurrent stroke	Contraindicated in pregnancy, risk of angioedema	Cough, hyperkalemia, hypotension
Angiotensin II Receptor Blockers (ARBs)	Comparable to ACE inhibitors with fewer respiratory complications	Contraindicated during pregnancy	Dizziness, hyperkalemia, rare cases of angioedema
Beta-Adrenergic Blockers	Indicated post-myocardial infarction, effective in heart failure	May exacerbate asthma, depressive symptoms	Bradycardia, cold extremities, fatigue, sexual dysfunction
Calcium Channel Blockers	Effective in older adults, beneficial for angina pectoris	Potential negative inotropic effects	Headache, constipation (verapamil), dizziness
Diuretics	Cost-effective, beneficial in older demographic	Electrolyte disturbances, frequent urination	Hypokalemia (thiazides), dehydration, gout, renal impairment
Alpha-1 Blockers	Beneficial for patients with concurrent benign prostatic hyperplasia	Initial dose hypotension	Dizziness, headache, palpitations
Direct Renin Inhibitors	Directly targets renin, renal protective effects	Limited long-term efficacy data	Diarrhea, dizziness, cough
Centrally Acting Agents	Effective for refractory hypertension	Potential for significant CNS effects	Drowsiness, sedation, dry mouth
Peripheral Adrenergic Inhibitors	Useful in specific hypertensive populations	Frequently limited by adverse effects	Orthostatic hypotension, reflex tachycardia
Vasodilators	Effective in severe hypertension	Can cause significant compensatory responses	Flushing, headache, fluid retention, tachycardia

β-blockers might be less effective in preventing strokes, especially in older patients, and have not been recommended by most international guidelines as a first line therapy for hypertension unless there is a compelling indication such as heart failure or cardiac arrhythmias. Also, β-blockers are associated with potential side effects such as weight gain, dyslipidaemia, and impaired glucose control. Therefore, individuals with or at risk of multiple metabolic issues may not be ideal candidates for β-blocker therapy unless they are using vasodilatory β-blockers, which are generally not linked with these adverse effects. Thiazide diuretics at high doses have also been implicated in dyslipidaemia and diabetes^26,27^. However, these effects are contested by results from the Antihypertensive and Lipid-Lowering Treatment to Prevent Heart Attack trial^28^.

## Effect of salt substitution on blood pressure control, cardiovascular events and death

The association between elevated dietary sodium consumption, as well as low levels of dietary potassium intake, to high blood pressure and an increased risk of cardiovascular disease and premature death is well established in literature. Randomized trials of dietary sodium reduction^29,30^, as well as trials of dietary potassium supplementation^31^, have shown clear blood-pressure–lowering effects. Salt substitutes, which replace part of the sodium chloride in regular salt with potassium chloride, combine these effects in a single product and have proven blood-pressure–lowering effects in diverse populations^31^. More recently, the Salt Substitute and Stroke Study (SSaSS), an open-label, cluster-randomized trial, conducted in China, included participants who had a history of stroke or were 60 years of age or older and had high blood pressure from villages randomly assigned in a 1:1 ratio to the intervention group, in which the participants used a salt substitute (75% sodium chloride and 25% potassium chloride by mass), or to the control group, in which the participants continued to use regular salt (100% sodium chloride). These trial showed that, among persons who had a history of stroke or were 60 years of age or older and had high blood pressure, the rates of stroke (29.14 events vs. 33.65 events per 1000 person-years; rate ratio, 0.86; 95% confidence interval [CI], 0.77–0.96; *P* = 0.006), major cardiovascular events (49.09 events vs. 56.29 events per 1000 person-years; rate ratio, 0.87; 95% CI, 0.80 to 0.94; *P* < 0.001), and death from any cause (39.28 events vs. 44.61 events per 1000 person-years; rate ratio, 0.88; 95% CI, 0.82 to 0.95; *P* < 0.001) were lower with the salt substitute than with regular salt^32^.

## Digital health and blood pressure control

Recent advances in technology and data science have introduced new perspectives on blood pressure management, emphasizing the roles of big data, artificial intelligence (AI), wearables, and personalized medicine^33,34^. Big data has revolutionized many aspects of healthcare, including stroke management. By aggregating vast amounts of patient data from electronic health records (EHRs), clinical trials, and real-world evidence, researchers and clinicians can identify patterns and correlations that were previously unobservable. These insights enable more accurate risk stratification, predicting which patients are at higher risk for complications, including secondary strokes, based on their blood pressure trends and other variables^35^.

For instance, big data analytics can uncover how different patient populations respond to various antihypertensive treatments, allowing for more targeted therapy. Additionally, integrating genetic, lifestyle, and comorbidity data helps develop comprehensive management plans tailored to individual patient needs^36^. One notable example is the use of AI to create predictive models that forecast blood pressure fluctuations and potential hypertensive crises. By analysing continuous blood pressure data alongside other health indicators, AI systems can alert healthcare providers to impending issues before they become critical^37^.

Furthermore, AI-powered decision support systems can recommend personalized treatment adjustments, considering the unique response patterns of each patient. These systems enhance the precision of blood pressure management, reducing the likelihood of adverse events and improving patient outcomes^35,38^. Wearable technology has become a game-changer in monitoring blood pressure and cardiovascular health. Devices such as smartwatches and fitness trackers equipped with blood pressure sensors allow continuous, non-invasive monitoring. This real-time data collection is invaluable for stroke survivors who require close monitoring to prevent secondary events^39^. They enable early detection through real-time alerts for abnormal readings, allowing immediate medical intervention to prevent complications. Additionally, wearables enhance patient engagement by encouraging adherence to treatment plans and lifestyle modifications.

In a similar direction, precision medicine is at the forefront of modern stroke care, aiming to tailor treatment plans to the individual characteristics of each patient. By leveraging big data and AI, precision medicine can identify the most effective interventions based on genetic profiles, biomarker analysis, and personal health data^38^.

In the context of blood pressure control, precision medicine entails crafting customized treatment plans by developing antihypertensive regimens that are tailored to an individual’s genetic makeup, comorbidities, and lifestyle factors. It also includes dynamic adjustments, meaning the continuous updating of treatment strategies based on new data to ensure alignment with the patient’s changing health status. Additionally, predictive analytics are used, employing AI models to forecast patient responses to specific treatments, which allows for proactive adjustments and enhanced outcomes^38,40^.

## Equity and inclusion in blood pressure control

Blood pressure control is critical, particularly among minority populations who are disproportionately affected by stroke and its risk factors. Several studies have explored various strategies and their effectiveness in managing blood pressure among these high-risk groups^41,42^.

Peer education has been investigated as a method to improve blood pressure control among minority stroke survivors. The Prevent Recurrence of All Inner-City Strokes through Education (PRAISE) trial, for instance, tested a peer-led, community-based stroke prevention program. The study found that while the intervention modestly improved blood pressure control, it did not significantly affect stroke risk factors such as low-density lipoprotein cholesterol or antithrombotic use. Another study highlighted the challenges in achieving optimal blood pressure control among black and Latino populations, despite the use of proven-effective cardiovascular medication^43^.

Racial and ethnic disparities in BP control post-stroke have been well-documented. A study conducted in Northern California found that black patients had poorer blood pressure control compared to their white counterparts, even when they had similar access to healthcare and were more likely to be on antihypertensive therapy^44^. Another study using data from the National Health and Nutrition Examination Surveys identified that women, non-Mexican Hispanics, blacks, diabetics, and older individuals were more likely to have poorly controlled blood pressure^45^.

Recruiting minority and low-income patients for blood pressure control trials poses significant challenges. The IMPACTS-MIND trial, which aimed to implement intensive blood pressure control strategies to slow cognitive decline, successfully recruited a diverse cohort but highlighted the need for improved methods to engage these populations.

## Perspectives and conclusion

Looking ahead, the future of managing blood pressure post-stroke is set to evolve towards more personalized treatment strategies. Technological advancements, including remote monitoring and telemedicine, are emerging as valuable tools to enhance treatment adherence and follow-up care. Furthermore, ongoing research into innovative antihypertensive therapies and combination treatments promises to refine blood pressure management tactics^42^. The Optimal Stroke Trial, a randomized controlled trial conducted in Brazil with 4,368 participants, aims to resolve prevailing uncertainties about the ideal blood pressure levels after a stroke (NCT04036409). The Reducing Blood Pressure in Patients With High Cardiovascular Risk in the Safety-Net (BP-REACH) aimed to evaluate the impact of an intervention on systolic blood pressure (SBP) in individuals with a history of stroke or MI through a randomized controlled trial in the Los Angeles Department of Health Services public healthcare system. The primary outcome was the change in SBP at 12 months. The study included participants from inpatient and outpatient settings in the Los Angeles County Department of Health Services health centers, randomized 1:1 to either usual care or the intervention. Outcomes were measured at baseline, 3 months, and 12 months, with the primary outcome being SBP (NCT05937685). The Intensive Blood Pressure Control in Ischaemic Stroke Patients With Severe Cerebral Small Vessel Disease (NCT05690997) hypothesizes lowering blood pressure (BP) to a SBP of 120–129 mmHg in ischemic stroke patients with severe small vessel disease (SVD) will not impair cerebral perfusion or worsen structural connectivity and cognitive function. This one-year trial included patients aged 50 and older with a history of ischemic stroke and severe cerebral SVD. Participants were be randomized (1:1) to a systolic BP target of either 120–129 mmHg or 130–140 mmHg. Participants underwent brain MRI at baseline and after one year to assess cerebral blood flow (CBF) and white matter integrity. They will also complete neuropsychological tests to evaluate cognitive function. Additionally, participants did monitor their BP at home, with periodic medication adjustments by a doctor to maintain the target BP. Finally, the Blood Pressure Reduction to Limit the Evolution of Vascular Brain Lesions in Elderly Individuals (LEOPOLD) trial is still recruiting patients (NCT02472028). The study aims to test the hypothesis that lowering blood pressure can slow the progression of White Matter Lesions (WML) in patients who have cognitive complaints and exhibit a moderate to high grade of WML on brain MRI. This PROBE (Prospective randomized open blinded end-point) trial involves a blinded reading of MRI scans for all patients in each group. After stratified randomization by age, sex, and center, patients will be assigned to one of two strategies: Reinforced Group (an enhanced strategy targeting a systolic BP of less than 135 mmHg) and Usual Group (a usual care strategy based on routine care practices).

In conclusion, while significant progress has been made in understanding and managing blood pressure post-stroke, ongoing efforts are required to refine treatment targets, overcome challenges, and implement effective, personalized care strategies. Collaborative efforts among healthcare providers, patients, and policymakers are crucial in overcoming current challenges and improving the quality of care and outcomes for stroke survivors. Through continued research and innovation, the goal of reducing recurrent strokes and improving the quality of life for stroke survivors can be progressively achieved.
